# The role of cognitive and brain reserve in the clinical presentation and progression of amyotrophic lateral sclerosis

**DOI:** 10.1038/s41598-025-97509-y

**Published:** 2025-06-20

**Authors:** Anna G. M. Temp, Gaël Nils Tarakdjian, Elisabeth Kasper, Judith Machts, Jörn Kaufmann, Stefan Vielhaber, Johannes Prudlo, James H. Cole, Martin Dyrba, Stefan Teipel, Andreas Hermann

**Affiliations:** 1 Department of Neurology, Translational Neurodegeneration Section “Albrecht-Kossel”, University Medical Center Rostock, University of Rostock, Gehlsheimer Straße 20, 18147 Rostock, Germany; 2 Deutsches Zentrum Für Neurodegenerative Erkrankungen (DZNE) Rostock/Greifswald, 18147 Rostock, Germany; 3 Center for Transdisciplinary Neurosciences Rostock (CTNR), University Medical Center Rostock, University of Rostock, 18147 Rostock, Germany; 4 Department of Neurology, University Medical Center Rostock, University of Rostock, 18147 Rostock, Germany; 5 Institute for Cognitive Neurology and Dementia Research, Otto-Von-Guericke University Magdeburg, 39120 Magdeburg, Germany; 6 Center for Behavioral Brain Sciences CBBS, 39104 Magdeburg, Germany; 7 Deutsches Zentrum Für Neurodegenerative Erkrankungen (DZNE) Magdeburg, 39120 Magdeburg, Germany; 8 Department of Neurology, Otto-Von-Guericke University Magdeburg, 39120 Magdeburg, Germany; 9 Centre for Medical Image Computing, Department of Computer Science, UCL, London, UK; 10 Dementia Research Centre, Queen Square Institute of Neurology, UCL, London, UK; 11 Department of Psychosomatic Medicine, University Medical Center Rostock, University of Rostock, 18147 Rostock, Germany

**Keywords:** Amyotrophic lateral sclerosis, Amyotrophic lateral sclerosis

## Abstract

Recent research has shown that cognitive reserve is associated with better cognitive abilities in ALS/MND, and that a slow brain ageing speed is associated with intact cognition in ALS. This study compares the effects of cognitive reserve and the predicted brain age difference (PAD) on the risk of being diagnosed with ALS, the risk of having cognitive or behavioral impairment, or even fronto-temporal dementia, and on disease duration.

Our results indicated that neither PAD nor cognitive reserve was associated with an increased risk of ALS, but that higher PAD was associated with an increased risk of cognitive impairments and FTD, as well as a shortened disease duration. Higher cognitive reserve on the other hand was associated with a lower risk of cognitive impairment and a longer disease duration.

Brain age as a proxy of brain reserve influences disease progression and presentation more strongly than cognitive reserve.

## Introduction

Cognitive reserve (CR) describes the theory that premorbid cognitive ability, educational and occupational attainment facilitate better coping with brain pathology, leading to individual differences in task processing^1^. CR is an active model of reserve because it posits that i) the brain actively uses pre-existing cognitive processes and/or ii) enlists compensatory strategies (^1^. Concerning Alzheimer’s disease (AD), high CR is associated with a relative risk reduction between 47–68%, depending on how CR is measured. Furthermore, evidence is mounting that bilingualism as a proxy of CR may delay symptom onset by several years^2^.

The concept of CR has recently been carried over from AD to diseases of the amyotrophic lateral sclerosis and fronto-temporal dementia spectrum (ALS-FTDSD)^3–7^. Work including structural and functional brain imaging techniques showed that persons with higher levels of education and premorbid cognitive ability were able to retain better cognitive functioning after the onset of ALS^3–5^. Over the course of a year, people living with ALS with higher cognitive reserve were able to retain better functioning, even against increasing brain atrophy^3^.

Additionally, our recent work has shown that people whose brains appeared younger than their chronological age were less likely to develop cognitive impairments alongside ALS-FTDSD^8^. Conversely, accelerated brain ageing was associated with FTD. The driving force behind the resilience against cognitive impairment appeared to be higher cerebellar volume. These findings are consistent with the brain reserve (BR) hypothesis: individual differences in people’s brains facilitate better coping with neurodegenerative diseases^1^. Such differences include brain volume (i.e., size) and synaptic density but also life experiences which influence brain anatomy via neurogenesis, angiogenesis, apoptosis and neural plasticity. BR is conceived as a passive model of reserve in the sense that a fixed amount of volume and synaptic density is assumed, alongside a threshold of maximum damage any given individual brain can cope with. In this model, BR is considered depleted once the threshold of maximum damaged has been crossed, and the clinical and/or functional deficits occur. The concept of brain age provides a proxy of BR, i.e. the difference between brain age estimated from MRI scans and chronological age (predicted age difference, PAD). PAD does not have an empirically established threshold of maximum damage, but our previous work suggests that a difference of six years is an appropriate threshold for cognitive impairment in ALS (ALSci)^8^. Notably, as cognitive impairment in ALS-FTDSD is considered a spectrum, not a continuum, it is not expected that all patients with ALSci will inevitably progress to ALS-FTD.

This present work will bring together the CR and BR hypotheses to investigate whether their protective effects, which long have been established in AD, do exist in the ALS-FTD spectrum diseases, too. This is distinct from our prior work which either excluded CR^8^ or excluded BR from its designs^3,4^.

Within the ALS-FTD spectrum, it has not yet been established whether cognitive and brain reserve have similar effects. The present study combines four potentially protective characteristics to form a measure of cognitive reserve: educational years, occupational level, presumed premorbid intelligence and PAD (analogue to Consonni et al.’s work,^7^). Using these, we addressed four research questions. Firstly, we will examine the probability of developing an ALS-FTD spectrum disease based on these potentially protective characteristics. Secondly, we will investigate the patients’ probability of being classified as cognitively or behaviourally impaired based on these characteristics. Thirdly, we will examine the effect of the potentially protective characteristics on patients’ survival time, controlling for Strong classification. The Strong criteria^9^ facilitate the classification of patients according to their non-motor impairments: cognitive impairment without dementia (ALSci), behavioural impairment without dementia (ALSbi), cognitive-behavioural impairment without dementia (ALScbi), and ALS with FTD (ALS-FTD) or primary progressive aphasia (ALS-PPA). Fourthly, we will compare their effects on age at disease onset.

## Methodology

### Design

We further analysed the data from our recent study on brain age in ALS (^8^). In brief, we drew our data from the *ALS-FTD Intersite* project, conducted at German Center for Neurodegenerative Diseases (DZNE) sites Rostock and Magdeburg, respectively. This observational, cross-sectional study ran between April 2011 and August 2013. Local ethics committees at both study sites approved the study (Rostock: A 2011 56; Magdeburg: 75/11). All participants provided written informed consent; the study was conducted in accordance with the Declaration of Helsinki. Data collection procedures are described by Kasper et al.^10^, while brain age estimation procedures are described by Hermann et al.^8^. The cohorts between this work and Hermann et al. are identical, with distinct research questions and analyses.

### Participants

In total, 178 participants were recruited from out-patient clinics and through public advertisement in Rostock and Magdeburg, Germany. Based on our selection criteria, persons with a history of brain injury, epilepsy, psychiatric illness or non-native command of the German language were excluded from participation in both, control group and patient group. ALS patients were only included if they underwent neuropsychological assessment and if data on education and premorbid-IQ was available. Control participants were further screened for cognitive impairment using the Montreal Cognitive Assessment and excluded if they scored below 26/30. The control cohort (n = 32) was age- and sex-matched to the patient cohort (n = 86). Complete data were available for 118 participants, their demographic details can be found in Table 1.

**Table 1:** Demographic background of the sample, stratified by Strong profile.

	HC	ALSni	ALSbi	ALSci	ALS-FTD
N	32	45	11	23	5
Age at onset	–	56.32 (9.76)	54.78 (13.74)	59.94 (11.15)	54.95 (10.93)
chronological age	61.07 (11.19)	59.20 (9.38)	57.90 (12.47)	63.34 (9.50)	56.59 (10.71)
brain age	58.86 (12.51)	55.69 (9.91)	56.80 (14.20)	66.67 (11.51)	62.56 (7.70)
PAD	-2.20 (5.72)	-3.54 (5.46)	-1.10 (3.64)	3.33 (5.95)	5.97 (10.64)
educational years	13.16 (1.69)	13.49 (2.75)	14.00 (2.00)	11.87 (1.71)	13.40 (1.67)
Premorbid IQ	104.41 (8.38)	103.00 (11.79)	102.91 (8.58)	93.57 (9.58)	96.00 (6.89)
Cognitive reserve	40.79 (3.00)	40.43 (4.89)	40.61 (3.33)	36.65 (3.36)	38.07 (2.64)
ALS-FRS-R	–	39.38 (6.30)	36.09 (7.53)	39.91 (5.08)	43.20 (2.77)
ALS-FRS-R delta	–	0.53 (0.40)	0.89 (0.92)	0.56 (0.36)	0.39 (0.21)
Disease Duration (months)	–	34.20 (23.94)	16.75 (12.90)	23.11 (13.28)	23.00 (19.09)
Sex (f/m)		27/18	7/4	16/7	4/1
Phenotype (PLS, PMA, UMN, classic, flail arm, flail leg, uncertain)	–	5/4/3/24/4/3/2	0/1/0/10/0/0/0	1/2/2/15/2/1/0	0/0/3/2/0/0/0
Onset Type (bulbar, limb, uncertain)	–	27/12/6	5/4/2	11/8/4	1/4/0

ALS patients were included if they showed progressive muscular weakness in ≥ body regions to also include ALS variants of upper and/or lower motor neuron predominance. ALS diagnoses were made using the revised El Escorial criteria^11^. Cognitive classification followed the Strong and Rascovsky criteria^9,12^, i.e., patients were classified into the following groups: ALS without cognitive-behavioural impairments (ALSni [not impaired]), ALS with cognitive impairment (ALSci), ALS with behavioural impairments (ALSbi), ALS with cognitive and behavioural impairments (ALScbi) and ALS with frontotemporal dementia (ALS-FTD).

Age at onset in this cohort falls within the recently reported onset between 51 and 66 years of age, with German patients reportedly developing the disease around 61 years of age^13^.

### MRI Acquisition

MRI scanning was performed with two 3T Siemens Magnetom VERIO scanners (Erlangen, Germany) using a 32-channel head coil; one single scanner at each site (Rostock and Magdeburg, Germany). High-resolution T_1_-weighted anatomical images were acquired using the magnetization-prepared rapid gradient echo (MPRAGE) sequence with the following parameters: 256 × 256 image matrix with 192 sagittal slices, FOV 250 × 250x192mm, voxel size 1 × 1x1mm^3^, echo time 4.82ms, repetition time 2500ms, and flip angle 7°.

### Measures

#### Cognitive reserve (CR)

This numerical variable was constructed as a composite measure with three proxies: educational years, educational attainment, and verbal intelligence as a measure of general intelligence. Verbal intelligence was measured by passive vocabulary^14^; for details see our previous work^3,4^. In ALS, performance on this specific test has been shown to remain intact and indistinguishable from healthy controls^15^, and independent from physical disability. We assigned points for each educational year, and according to the international standard classification of education (ISCED) to reflect educational length and achievement. Crucially, the former – educational years – assigns points purely based on the length of time spent in formal education while the latter – ISCED levels – reflects educational achievement. For example, it is possible to obtain 13 years of education by primary and secondary education (“Realschule”) followed by 3 years of vocational training (“Berufsausbildung”), resulting in ISCED level 4, or instead of “Realschule” by completing secondary education (“Gymnasium”) without any further tertiary education or vocational training –resulting in ISCED level 3. Our CR measure was the mean of educational years, ISCED level and IQ points. Consequently, a numerically higher value indicates a higher level of cognitive reserve.

*Chronological age.* Age from birth to the time of data collection, rounded to full years.

*Predicted age difference (PAD).* The individual deviation between estimated brain age and chronological age, in years. Persons whose brains appear older than their chronological age will have positive values, for example if a 60-year-old’s brain is estimated to be 65 years old, their PAD would be 5. Consequently, persons whose brain appear younger than their chronological age will have negative values, if a 60-year-old’s brain is estimated to be 55 years old, then their PAD would be -5. PAD serves as our proxy of BR, with persons who have a numerically high PAD and therefore an older brain conceptualized as having “low brain reserve” and those who have a numerically lower value thought to have “high brain reserve”.

Brain age model and predicted brain age difference (PAD): We estimated brain age in R, using the package “brainageR” (version 2.1), available at https://github.com/james-cole/brainageR. This algorithm was trained on n = 3377 healthy adults and validated on 857 people. To predict brain age, we followed an automated pipeline starting with T1-weighted image segmentation and normalization using SPM12 with smoothing with a 4 mm Gaussian kernel and modulation to match with the training sample. Then, the spatially normalized grey and white matter and cerebrospinal fluid probability maps were loaded into R and vectorised. They were masked to exclude voxels with less than 30% probability for the corresponding brain tissue class. Subsequently, these maps underwent a principal component analysis transformation, based on the training data. The transformed data were then entered in the pretrained Gaussian process regression model to obtain the predicted brain age. Finally, chronological age was subtracted from the brain age to calculate PAD.

*Strong profile.* Participants underwent comprehensive neuropsychological assessment, as described previously^16,17^, and classified according to the Strong criteria^18^. Diagnoses of ALS-FTD were made based on the Rascovsky criteria^12^.

*ALS vs HC.* This binary variable grouped all ALS patients together, regardless of non-motor impairment, to distinguish them from HC.

### Statistical analysis

All statistical modelling took place using the *brms* package in R. Predictors included CR, PAD, age, sex and Strong profile. The outcome variables were group (ALS vs HC), Strong profile, total disease duration, and age at onset. Strong profile served as outcome for our second research question, and as a predictor in our third and fourth research question.

The focus of our work was parameter estimation, so individual predictors’ significance is determined by their 95% credible intervals: credible intervals (CI) which contain zero indicate that zero is a plausible effect size for the predictor under consideration. Consequently, predictors with a plausible effect size of zero were not considered significant. In the case of odds ratios (OR), CI overlapping 1 were not considered significant. To determine significance, we used results based on standardized outcomes; the interpretation of these results however relied on unstandardized variables.

To examine the probability of being classified as having an ALS-FTD spectrum disease instead of as a healthy control person based on these potentially protective characteristics, we chose a logistic regression using the Bernoulli distribution with the predictors of age, sex, CR and PAD on the binary outcome variable “HC vs ALS-FTDSD”. To examine the effect of PAD and CR on the probability of an ALS patient developing cognitive-behavioural impairments, we conducted a multinomial regression using the “categorical” family; the predictors were age, sex, CR and PAD while Strong criteria classification served as the outcome. The effects on patients’ survival and age at onset were modelled with Gaussian distributions. We specified subjective priors reflecting our hypotheses and expectations as outlined in the introduction; these priors including their justification can be found in the online supplement, see Data Availability Statement. Multicollinearity was assessed using the *performance* package, with the function check_collinearity().

## Results

**Fig. 1:**
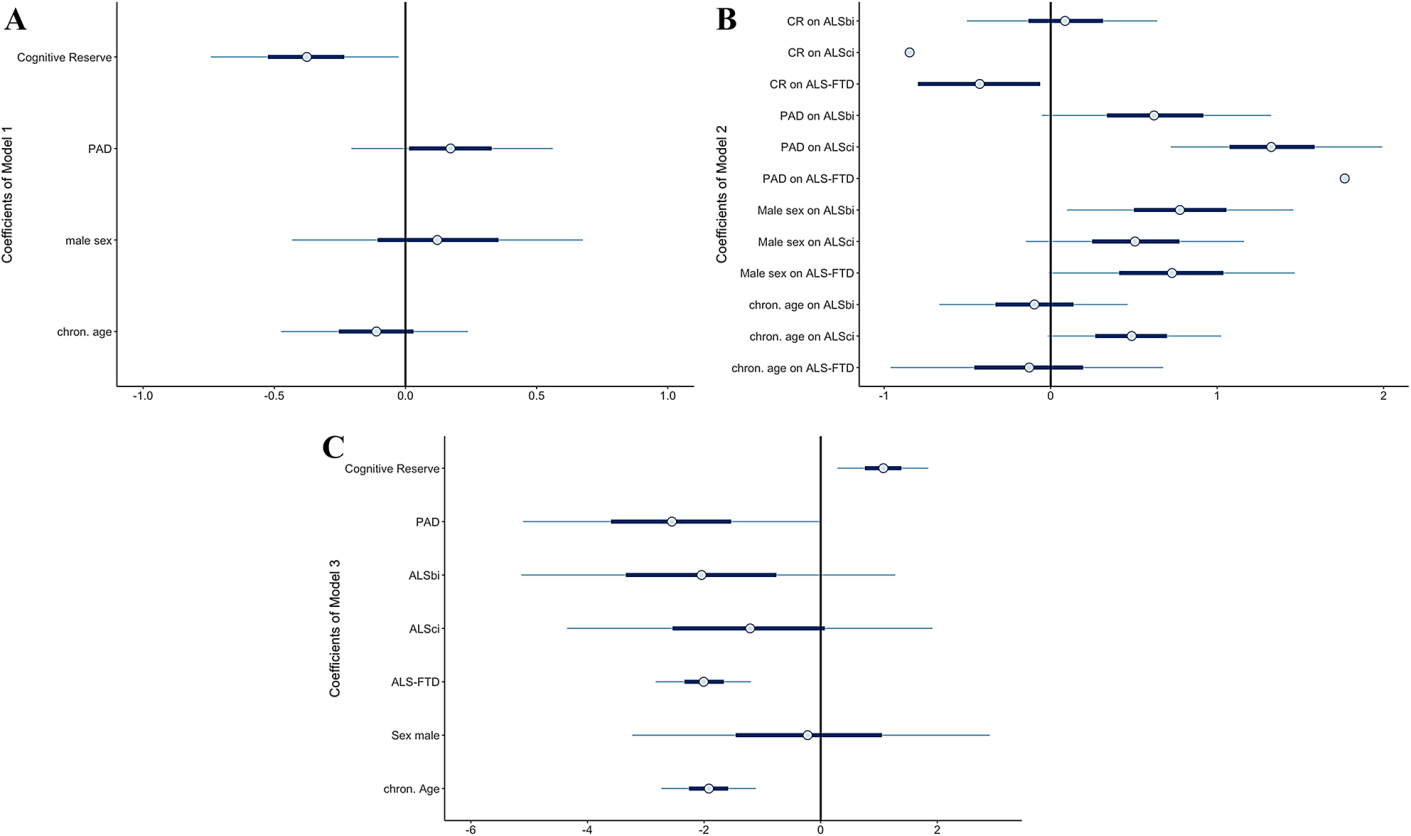
(**A**). The coefficients of model 1(including credible intervals). Notably, all predictors’s CI overlap or include 0, indicating that it is plausible that CR, PAD, male sex and age do not have any effect on the probability of being diagnosed with ALS. Figure 1 (**B**). The coefficients of model 2 (including credible intervals): PAD increases the probability of being diagnosed with behavioural impairments (ALSbi), cognitive impairments (ALSci) or ALS-FTD. CR, however, lowers the risk of being diagnosed with ALSci. Figure 1 (**C**). The coefficients of model 3 (including credible intervals): PAD, a diagnosis of ALS with behavioural impairments or FTD are associated with a shorter disease duration, while higher CR is associated with a longer disease duration.

We explored four research questions. Firstly, we examined the odds ratios (OR) of developing an ALS-FTDSD based on CR and PAD (Model 1, Fig. 1A). Secondly, we investigated the OR of being classified as cognitively or behaviourally impaired based on CR and PAD (Model 2, Table 2, Fig. 1B). Thirdly, we examined the effect of PAD and CR on patients’ survival time, controlling for Strong classification (Model 3, Table 2, Fig. 1C). Fourthly, we explored any associations between CR, BR and age of disease onset (Model 4, Table 2). The OR, standardised (β) and unstandardised (B) coefficients of the significant predictors can be obtained from Table 2 below. Details on non-significant coefficients can be found in our online supplement, see Data Availability Statement. Please note that Model 1 resulted in no significant effects at all; hence, it is excluded from Table 2 and Fig. 1.

**Table 2:** Odds ratios, standardised and unstandardised coefficients of the significant predictors.

Outcome	Effect	OR (95%CI)	B (95%CI)	β (95%CI)
Strong criteria	PAD on ALSci	1.24 (1.11|1.42)	0.22 (0.10|0.35)	1.33 (0.61|2.13)
	CR on ALSci	0.82 (0.67|0.98)	− 0.20 (− 0.40|-0.02)	− 0.86 (1.58|-0.18)
	PAD on ALS-FTD	1.36 (1.13|1.69)	0.31 (0.12|0.53)	1.77 (0.64|2.97)
Disease Duration	Age	–	− 0.55 (− 1.19|-0.00)	− 1.93 (-2.84|-1.02)
	ALS-FTD	–	− 2.01 (− 2.99|-1.03)	− 2.01 (− 2.97|-1.07)
	CR	–	0.67 (− 0.13|1.43)	1.06 (0.14|2.04)
Age at Onset	Age	–	1.00 (0.91|1.09)	4.23 (3.18|5.27)

OR: Odds ratios based on unstandardized coefficients, B: unstandardized coefficient; β: standardized coefficient; CI: credible interval

Multicollinearity was low, across all models and variables, with the variance inflation factors (VIF) well below 5.

Figure 1 illustrates the standardized predictors and their CI.

**Research question 1:** How do PAD and CR affect the probability of being diagnosed with ALS altogether?

This logistic regression used the Bernoulli distribution as a prior with the predictors of age, sex, CR and PAD on the binary outcome variable “HC vs ALS-FTDSD”. There were no meaningful effects as all OR derived from standardized coefficients overlapped 1, indicating that there was great uncertainty in our data as to whether PAD (OR = 1.19, CI: 0.76–1.87), CR (OR = 0.68, CI: 0.45–1.05), chronological age (OR = 0.89, CI: 0.59–1.35) and sex (OR = 1.13, CI: 0.59–2.20) affect the probability of developing ALS. This was also true for OR derived from unstandardized coefficients. Consequently, these data do not provide any evidence for or against the effect of PAD and CR on the probability of developing ALS, see . 1A.

**Research question 2:** Once ALS is diagnosed, do PAD and CR affect the probability of being diagnosed with cognitive-behavioural impairments?

This multinomial regression used the “categorical” family, with the predictors age, sex, CR and PAD with Strong criteria classification as the outcome. Patients with higher PAD had a higher risk of being diagnosed as ALSci or ALS-FTD (Table 2, . 2A). For each additional year in PAD, the risk of being diagnosed as ALSci increased by 24%, and the risk of being diagnosed as ALS-FTD increased by 36% (see the OR in Table 2).

**Fig. 2:**
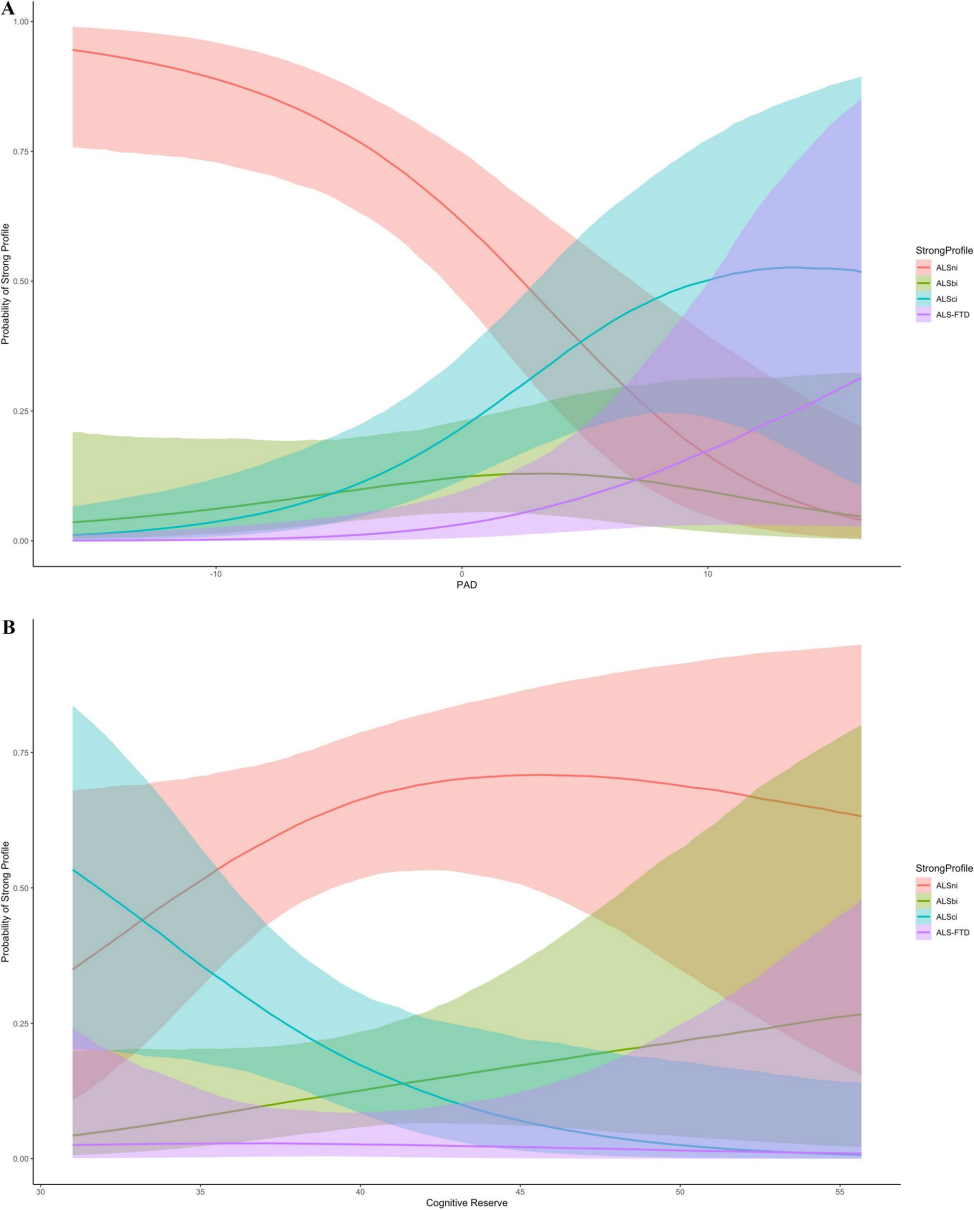
(**A**): The effect of PAD on the probability of being diagnosed with the respective Strong profile. The probability of having no cognitive or behavioural impairment decreases with increasing PAD, while the probability of having cognitive or behavioural impairment as well as FTD increases when PAD is positive (i.e., the brain is estimated to be older). Figure 2 (**B**): The effect of CR on the probability of being diagnosed with the respective Strong profile. The probability of having ALS and no cognitive nor behavioural impairment increases with increasing CR, while the probability of having ALS with cognitive or behavioural impairment decreases drastically when CR increases.

Conversely, patients with higher CR had a lower risk of being diagnosed with ALSci (Fig. 2B). For each point in CR, the probability of being diagnosed as ALSci decreased by 18%. These standardized coefficients indicate that the detrimental effect of PAD is stronger than the beneficial effect of CR.

There were no unidirectional effects of ALS or CR on the risk of being diagnosed as ALSbi, and no effects of sex or chronological age on any Strong profile (Fig. 1B, Table 2).

Since cognitive impairment is reported to be influenced also by onset type, we explored onset type as a covariate in this analysis. Onset type did not significantly predict outcomes, as the 95% credible intervals for its effects encompassed zero (ALSbi: b = –0.59, 95% CI: –2.29 to 1.06; ALSci: b = –0.47, 95% CI: –1.95 to 1.01; ALS‐FTD: b = –2.32, 95% CI: –5.22 to 0.04). When onset type was included, only the effects of PAD remained robust, showing significant associations in ALSci (b = 1.20, 95% CI: 0.41 to 2.09) and ALS‐FTD (b = 1.60, 95% CI: 0.42 to 2.88), while the effect of CR was no longer significant.

**Research question 3:** How do Strong profile, PAD and CR affect total disease duration?

**Fig. 3:**
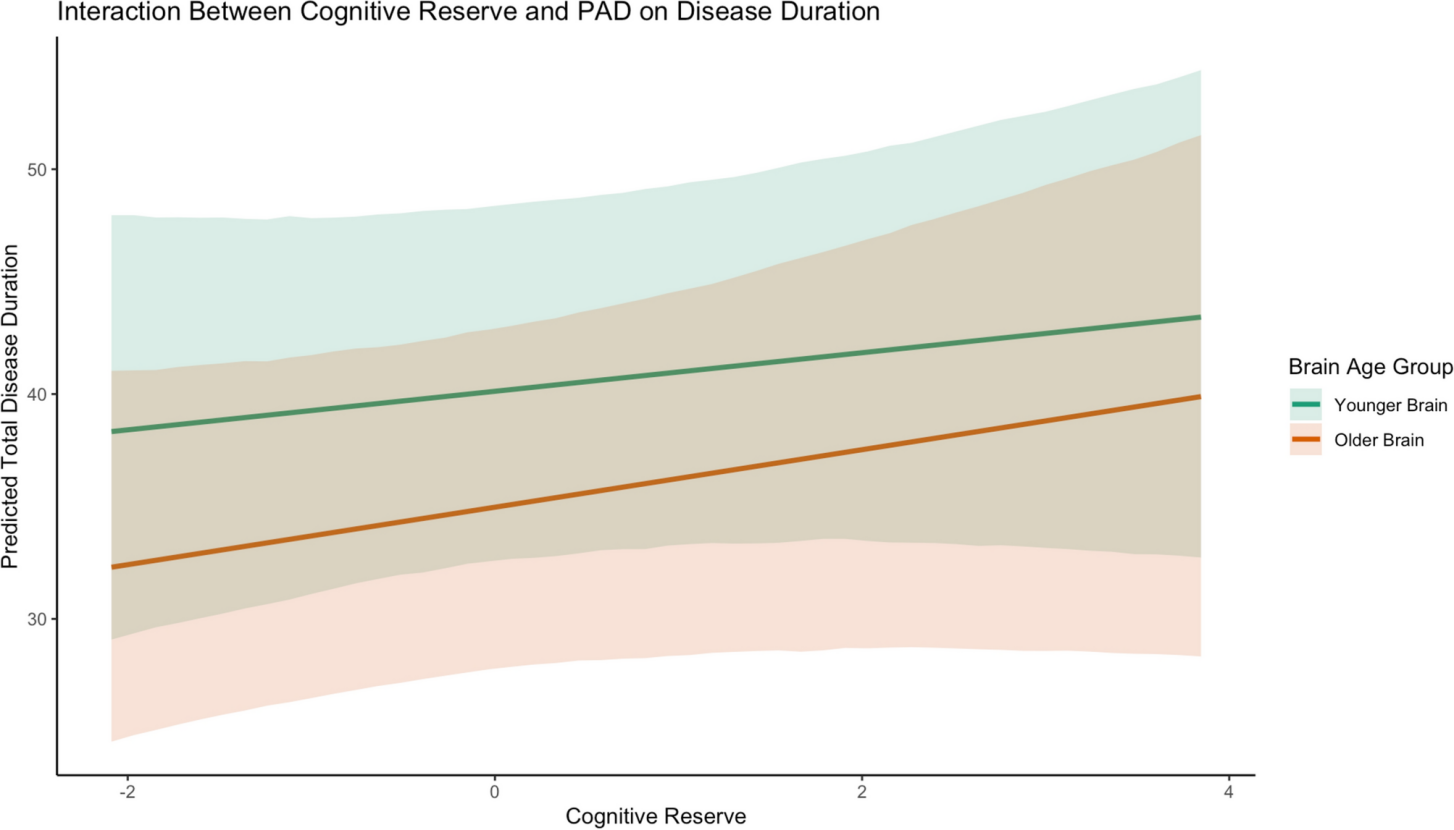
Interaction Between Cognitive Reserve and Predicted Age Difference (PAD) on Total Disease Duration. The figure illustrates the relationship between CR and total disease duration, stratified by brain age group. Patients were categorized as having a “Younger Brain” (green line; PAD < 0) or “Older Brain” (orange line; PAD > 0), based on whether their predicted brain age was younger or older than their chronological age, respectively. The plotted regression lines indicate that higher CR is associated with longer total disease duration in both groups. However, the slopes remain nearly parallel, suggesting that the association between R and disease duration is not significantly moderated by PAD. Shaded areas represent 95% credible intervals, which overlap substantially between groups, further supporting the absence of a meaningful interaction effect. Notably, the y-axis intercepts indicate that, at the lowest observed levels of CR, patients with an older brain tend to have a shorter predicted disease duration than those with a younger brain. These findings indicate that while both CR and PAD may influence disease duration, their effects appear to be largely independent.

This was a linear regression assuming a Gaussian distribution, with CR and BR as the predictors, age and sex as the covariates and months of total disease duration as outcome. Among all patients, those with higher CR lived longer: each 1-point increase in CR was associated with 0.67 additional months of lifetime (approximately 20 days, see the OR in Table 2). Conversely, being diagnosed with ALS-FTD decreased patients’ lifetime by two months (b = -2.01, Table 2). A detrimental effect was also observed for chronological age at time of assessment, for each additional year of age at diagnosis, participants lived approximately 17 days shorter (b = -0.55). There were no effects of PAD, sex, ALSbi or ALSci on total disease duration (see Fig. 1C). We re-ran this analysis excluding CR as a predictor to be able to better compare it to our previous work which relied on a simple Kendall’s tau correlation^8^. This linear regression supported a negative effect of PAD on disease duration (b = -1.09, 95%CI -2.03 to -0.20). This intriguing observation prompted us to explore the association between PAD and CR in their effects on disease duration more thoroughly. As reported above, their VIF were well below 5 (VIF_CR_ = 1.00, VIF_PAD_ = 1.02), indicating that multicollinearity between them was low. We re-ran the linear regression with PAD and CR as predictors and added their interaction term. However, the interaction term yielded no meaningful effect (b = -0.20, 95% CI: -1.70 to 2.05), suggesting that CR does not systematically alter the influence of PAD on disease duration. This conclusion is supported by Fig. 3, where we grouped patients into “Older Brain” (PAD > 0) and “Younger Brain” (PAD < 0). The nearly parallel slopes and substantial overlap in credible intervals indicate that the relationship between CR and disease duration remains consistent across brain age groups, reinforcing the absence of a meaningful interaction.

To complement this analysis, we explored the fact that persons with a faster disease progression speed would also die sooner. We investigated the effects of PAD and CR on disease progression speed, calculated as the delta of ALSFRS-R between reported symptom onset and first study visit (see Table 1). The predictors were PAD, CR, chronological age and sex. However, only chronological age turned out to be associated with disease progression speed (b = 0.18, 95% CI: 0.06 to 0.30), indicating that older patients showed a faster disease progression.

**Research question 4:** How do PAD and CR affect age at disease onset?

This was a linear regression assuming a Gaussian distribution, with CR and BR as the predictors, age and sex as the covariates and age at onset in years as outcome. None of the standardized coefficients suggested a meaningful effect of PAD or CR which is why we excluded these results from Figs. 1 and 2.

Finally, we also explored the fit of non-linear modelling techniques by fitting generalized additive models (GAM) in *brms* by adding smoothed terms for PAD and CR to each of the above models. We evaluated model fit using leave one out (LOO) cross-validation with the LOO information criterion. However, there was no significant difference in the fit of the GAM compared to the linear models outlined above, implying that there were no substantial non-linear or threshold effects present, and that the linear specifications adequately captured the relationships between PAD, CR, and the outcome variables.

## Discussion

We investigated and compared the effects of PAD and CR on the probability of developing ALS altogether, the probability of being classified as ALSni instead of being classified as ALSci, ALSbi, ALScbi and ALS-FTD, total disease duration and age of onset. The aim was to compare the effects of cognitive reserve as well as PAD, the latter as a proxy of brain reserve. Neither measure mediated the risk of being diagnosed with ALS altogether. However, with each additional year that their brain was estimated to be older than their chronological age, patients’ risk of being diagnosed with ALSci increased by 25%, and the risk of being diagnosed as ALS-FTD increased by 36%. Conversely, with each point in CR, their risk of being diagnosed as ALSci decreased by 20%. Similar protective effects by CR were observed on total disease duration: for each point in CR, patients’ lifespan was extended by 20 days. This effect size sounds trivial but when we consider that the range of CR in our sample was 31–56 points, the possible life span extension associated with CR ranged between 620 to 1120 days (20 to 37 months). Most notably, this lifestyle effect exceeds the effect of Riluzole treatment, for which randomised clinical trials suggested a lifespan extension of approximately three months, and whose real-world evidence suggests an extension of up to 19 months’ median time^19^. This highlights that CR supports patients’ ability to cope with the increasing load of ALS-related neuropathology as the disease progresses. Conversely, being diagnosed with ALS-FTD reduced patients’ disease duration by two months, i.e., these persons died two months sooner. Cognitive impairment being associated with a shorter lifespan is a well-documented effect in ALS-FTDSD^20,21^.

PAD not being associated with longer disease duration deviates from our own previous findings: we initially had observed a correlation whereby younger brain age correlated with longer disease duration. However, this previous finding was based on Kendall’s tau correlations, which did not feature confounding variables. The present analysis is a regression analysis which considered CR, sex and age in addition to PAD as predictors of disease duration. This prompts the discussion of why PAD is associated with an increased risk of ALS-FTD – which in turn reduces disease duration – without affecting disease duration directly. PAD only constitutes a crude indicator of brain health while ALS-FTD as a disease affects the brain as well as the spinal cord, and in turn, body regions beyond, which perhaps better reflects the disease in total. One possible explanation for this discrepancy is that PAD is strongly driven by cerebellar volume^8^, which is not a primary determinant of disease duration in ALS. While cerebellar atrophy has been linked to neurodegeneration and motor dysfunction, ALS progression is characterized by widespread involvement of both upper and lower motor neurons, as well as cognitive decline in ALS-FTD. Thus, PAD may capture neuroanatomical changes that increase susceptibility to ALS-FTD but do not directly regulate the speed of disease progression.

While work on CR and PAD and how they might affect disease outcomes in ALS is just at its beginning, this is well established in other neurodegenerative diseases, mainly in Alzheimer’s disease (AD). The concept of CR refers to the capacity to retain adequate cognitive functioning in the presence of neurodegenerative changes, while of brain aging refers to the preservation overall age-related brain atrophy. In AD, PAD was found to be associated with cognitive decline^22^ and molecular markers of pathology^23^. High CR may mitigate the accumulation rate of amyloid prior to cognitive impairment and support glucose metabolism during the dementia stage throughout the progression of AD pathology^24^.

CR not being associated with the risk of ALS, nor with a later disease onset distinguishes ALS from other neurodegenerative diseases such as AD, where its protective effects have long been documented^2,24–26^. Given that our analyses showed no effect of CR on disease progression speed, we do not think that ALS’ relatively aggressive progression is the reason for the absence of CR’s effect here. Rather, we hypothesise that the neuropathology underlying ALS predominantly accumulates outside of areas where CR typically exerts its protective influence, e.g. the motor cortex. It is also possible that CR influences early, pre-symptomatic stages of ALS that were not captured in this study. With our data also rejecting PAD as a protective factor, there is now evidence supporting the theory that CR and brain reserve affect patients’ ability to cope with neurodegenerative diseases distinctively. CR and PAD both mediating the probability of cognitive impairment, with PAD also playing a role in ALS-FTD is consistent with our prior work which showed that increased brain age was associated with fronto-temporal atrophy^8^, and that CR protects ALS patients from functional decline even when atrophy is present and increasing^3–5^. Additionally, since a lower PAD is partly driven by cerebellar volume^8^, this further distinguishes its effects from CR, which is more strongly associated with cortical and functional resilience. This difference may explain why PAD contributes to ALS-FTD risk but does not significantly influence disease duration once CR is included in the model.

A recent study investigated the interplay of CR and brain age and found that higher CR was associated with lower cognitive impairment independent of brain aging^27^, while lifestyle trajectories were associated with brain aging but independent of cognition^28^. This aligns closely with our findings that CR and PAD might predict different aspects of ALS disease outcome. Further work is needed to better understand this relationship, particularly in ALS.

Limitations of this study include the small sample sizes across the ALSbi and ALS-FTD subgroups, specifically, due to the relative rarity of the disease subtypes. Furthermore, there are some proxies of CR that were not available to us, such as level of regular exercise and multilingualism^7^. Furthermore, the brain-age analysis pipeline yields a single value and its ease of use might make it well suited in routine clinical care. However, it is conceptualized on the whole brain and distinct neuroanatomical information are not available. Consequently, it might not be sensitive enough for every disease entity depending on the spatial patterns of brain atrophy.

All in all, our findings provide some support for both, the cognitive reserve hypothesis and the brain reserve hypothesis. Given the effect sizes estimated here, it appears that CR had only moderately beneficial effects: namely, on the risk of developing cognitive impairment and longer disease duration. Contrastingly, the effect of accelerated brain ageing was more strongly detrimental, in the form of an increased risk of developing ALS-FTD. The finding that ALS-FTD is associated with a shorter disease duration independently from brain ageing speed supports the notion that not all factors underlying rapid disease progression are yet captured by brain age algorithms.

In conclusion, the present data indicate that the effect of low brain reserve is more detrimental than the positive effect of high cognitive reserve. However, both mechanisms appear to affect disease presentation and progression independently, and differently.

## Data Availability

The data in .csv file format, R code as an RMarkdown file and a full data report rendered into PDF are available from the Open Science Framework at: https://osf.io/td2xa/ . The code supports all the analyses and figures produced for this manuscript.
